# Expression and Clinical Significance of Androgen Receptor in Triple-Negative Breast Cancer

**DOI:** 10.3390/cancers9010004

**Published:** 2017-01-06

**Authors:** Yuka Asano, Shinichiro Kashiwagi, Wataru Goto, Sayaka Tanaka, Tamami Morisaki, Tsutomu Takashima, Satoru Noda, Naoyoshi Onoda, Masahiko Ohsawa, Kosei Hirakawa, Masaichi Ohira

**Affiliations:** 1 Department of Surgical Oncology, Osaka City University Graduate School of Medicine, 1-4-3 Asahi-machi, Abeno-ku, Osaka 545-8585, Japan; asnyk0325@yahoo.co.jp (Y.A.); saraikazemaru@gmail.com (W.G.); spitz4_5@yahoo.co.jp (T.M.); tsutomu-@rd5.so-net.ne.jp (T.T.); s-noda@med.osaka-cu.ac.jp (S.N.); nonoda@med.osaka-cu.ac.jp (N.O.); hirakawa@med.osaka-cu.ac.jp (K.H.); masaichi@med.osaka-cu.ac.jp (M.O.); 2 Department of Diagnostic Pathology, Osaka City University Graduate School of Medicine, 1-4-3 Asahi-machi, Abeno-ku, Osaka 545-8585, Japan; m1153321@med.osaka-cu.ac.jp (S.T.); m-ohsawa@med.osaka-cu.ac.jp (M.O.)

**Keywords:** triple-negative breast cancer, androgen receptor, prognostic marker, individualized treatment, intrinsic subtype

## Abstract

Background: Triple-negative breast cancer (TNBC) has a poor prognosis because of frequent recurrence. Androgen receptor (AR) is involved in the pathogenesis of breast cancer, but its role is not clearly defined. The aim of this study was to explore the expression of AR and its relationship with clinicopathologic features in TNBC. Methods: This study investigated 1036 cases of sporadic invasive breast carcinoma. Immunohistochemical assays were performed to determine the expression of AR in 190 TNBC samples. The relationships between AR expression and clinicopathologic data and prognosis were analyzed. Results: In 190 TNBC cases, the prognosis of AR-positive patients was significantly better (*p* = 0.019, log-rank) than AR-negative patients, and in multivariate analysis, AR expression was an independent indicator of good prognosis (*p* = 0.039, hazard ratio = 0.36). In patients with disease relapse, AR positivity was significantly correlated with better prognosis (*p* = 0.034, log-rank). Conclusions: AR expression may be useful as a subclassification marker for prognosis in TNBC.

## 1. Introduction

Breast cancer is a highly diverse disease that can be classified into subtypes comprising different clinical or cellular characteristics. It is commonly subclassified into five subtypes including luminal A, luminal B, human epidermal growth factor receptor 2 (HER2)-enriched, basal-like, and normal-like, according to the mRNA expression profile; these breast cancer types are frequently referred to as the “intrinsic subtype” [1,2,3,4]. The basal-like subtype almost always coincides with estrogen receptor (ER)-negative, progesterone receptor (PR)-negative, and HER2-negative triple-negative breast cancer (TNBC) [3,5,6]. However, compared to the basal-like classification that is based on a molecular approach, classifying a tumor as a TNBC is based on an immunohistochemical approach that is easy to use in actual clinical practice. TNBC is an intractable breast cancer because of its highly malignant biological potential, including aggressive tumor growth and rapid dissemination to important organs [7,8,9]. Patients with TNBC often require systemic anti-cancer therapy to manage the progression of the disease. Endocrine and anti-HER2 therapies are ineffective against TNBC as they lack the molecular targets (ER and HER2, respectively), and chemotherapy is considered the only remedy for TNBCs [10,11].

However, recent research indicates that TNBC can be further classified according to its genetic profile. Androgen receptor (AR)-positive TNBC is one of these subtypes [12]. AR-positive TNBC shows preserved androgenic signaling that could be a possible therapeutic molecular target similar to ER-positive breast cancer [10,13]. Additionally, AR expression has been identified in 70%–90% of breast tumors, similar to the frequency of ER expression in breast tumors [14]. Although previous reports indicated that androgens inhibit the progression of breast cancer [15,16,17], the precise mechanisms and clinical significance of AR in breast cancer remain unclear. International phase II studies aiming to develop a novel individualized treatment strategy against TNBC are currently underway in AR-positive TNBC [13]. There have been several reports investigating the clinical features of AR-positive TNBC [10,18,19,20,21,22]; most have found non-aggressive characteristics with a favorable prognosis compared with AR-negative TNBCs [18,22,23]. However, some reports have suggested positive correlations between AR positivity and progressive disease or poor prognosis [19]. Thus, controversies still exist concerning the clinical significance of AR expression in TNBC [24].

In this study, we classified 190 cases of breast cancer with the triple-negative phenotype from 1036 breast carcinomas. We addressed the significance of clinicopathologic features and AR expression in order to identify additional prognostic markers that can help identify tumors with more aggressive behavior.

## 2. Materials and Methods

### 2.1. Patient Background

This study investigated a consecutive series of 1036 patients with primary infiltrating breast cancer who underwent operations at the Osaka City University Hospital from 2000 to 2006. Additionally, 190 patients with TNBC treated at the Osaka City General Hospital were included. All of the patients who had undergone conservative breast surgery received postoperative radiotherapy to the residual breast. TNBC patients received adjuvant chemotherapy by either an anthracycline-based regimen (doxorubicin or epirubicin) or a 5-fluorouracil (5-FU)-based regimen, depending on the stage or risk of recurrence, in accordance with the National Comprehensive Cancer Network guidelines or the guidelines for breast cancer in Japan. The median follow-up time was 6.6 years (range, 0.2–8.0 years). Relapse-free survival (RFS) was defined as the interval between the date of surgical removal of the primary tumor and the date at which relapse was confirmed or the date of the last follow-up (for censored patients). Cancer-specific survival (CSS) was the time, in years, from the date of the primary surgery to the time of breast cancer-related death. Tumors were confirmed histopathologically and staged according to the TNM classification. This research conformed to the provisions of the Declaration of Helsinki in 1995. All patients were informed of the investigational nature of this study and provided their written, informed consent. The study protocol was approved by the Ethics Committee of Osaka City University (#926).

### 2.2. Immunohistochemistry

Immunohistochemical studies were performed as previously described [25]. The tumor specimens were fixed in a 10% formaldehyde solution and embedded in paraffin, after which they were cut into 4-µm thick sections and mounted on glass slides. The slides were deparaffinized in xylene and heated in an autoclave for 20 min at 105 °C and 0.4 kg/m^2^ in Target Retrieval Solution (Dako, Carpinteria, CA, USA). The specimens were then incubated with 3% hydrogen peroxide in methanol for 15 min to block endogenous peroxidase activity, and then incubated in 10% normal goat or rabbit serum to block nonspecific reactions.

Primary monoclonal antibodies directed against ER (clone 1D5, dilution 1:80; Dako), PR (clone PgR636, dilution 1:100; Dako), HER2 (HercepTest™; Dako, Carpinteria, CA, USA), Ki67 (clone MIB-1, dilution 1:100; Dako), and AR (clone AR441, dilution 1:100; Dako) were used. The tissue sections were incubated with antibody for 70 min at room temperature or overnight at 4 °C (HER2: 70 min; ER, PgR, Ki67, AR: overnight), and were then incubated with horseradish peroxidase-conjugated anti-rabbit or anti-mouse Ig polymer as a secondary antibody (HISTOFINE (PO)™ kit; Nichirei, Tokyo, Japan). The slides were subsequently treated with streptavidin–peroxidase reagent and incubated in phosphate-buffered saline-diaminobenzidine and 1% hydrogen peroxide (v/v), followed by counterstaining with Mayer’s hematoxylin. Positive and negative controls for each marker were used according to the supplier’s data sheet.

### 2.3. Immunohistochemical Scoring

**Figure 1:**
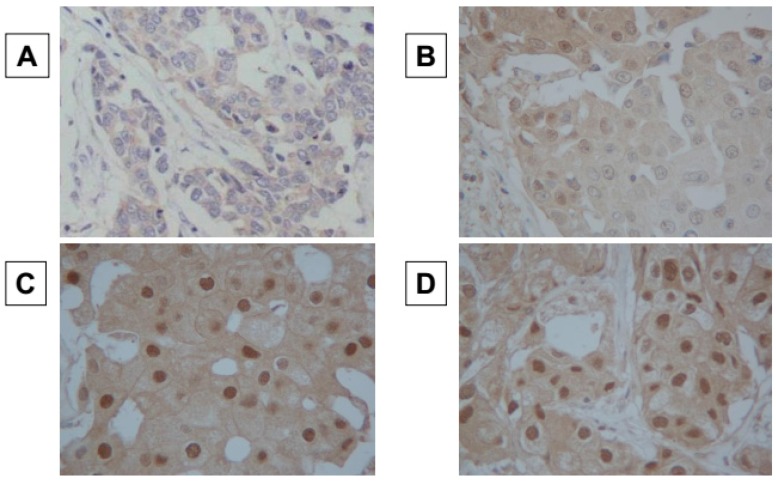
Immunohistochemical determination of androgen receptor. Androgen receptor (AR) expression was semi-quantitatively analyzed according to the percentage of cells showing nucleus tipositivity: 0, 0% (**A**); 1+, 1%–29% (**B**); 2+, 30%–69% (**C**); 3+, ≥70% (**D**). AR expression was considered positive when scores were ≥1, and negative when scores were 0. (×400).

Immunohistochemical scoring was performed by two pathologists who specialized in mammary gland pathology using the blind method to confirm the objectivity and reproducibility of the diagnosis. In line with previous studies, the cut-off value for ER and PR positivity was set at ≥1%, and the same cut-off was adopted for AR positivity. HER2 expression was graded according to the accepted grading system as 0, 1+, 2+, or 3+. The following criteria were used for scoring: 0, no reactivity or membranous reactivity in <10% of cells; 1+, faint/barely perceptible membranous reactivity in ≥10% of cells or reactivity in only part of the cell membrane; 2+, weak to moderate complete or basolateral membranous reactivity in ≥10% of tumor cells; 3+, strong complete or basolateral membranous reactivity in ≥10% of tumor cells. HER2 was considered positive if the grade of immunostaining was 3+, or 2+ with gene amplification via fluorescent in situ hybridization (FISH). In the FISH analysis, each copy of the HER2 gene and a reference gene (centromere 17; CEP17) was counted. The interpretation followed the criteria of the American Society of Clinical Oncology **(**ASCO**)**/College of American Pathologists **(**CAP**)** guidelines for HER2 immunohistochemistry classification for positive breast cancer if the HER2/CEP17 ratio was higher than 2.0 [26]. A Ki67-labeling index of ≥14% was classified as positive [27]. Immunohistochemical scoring of AR expression was evaluated as previously described [28,29,30]. AR expression was semi-quantitatively analyzed according to the percentage of cells showing nuclear positivity: 0, 0%; 1+, 1%–29%; 2+, 30%–69%; 3+, ≥70%. Scores ≥1 were considered positive, and a score of 0 was negative (Figure 1) [28,29,30].

### 2.4. Statistical Analysis

Statistical analysis was performed using the SPSS^®^ version 19.0 statistical software package (IBM, Armonk, New York, NY, USA). Categorical data are reported with numbers and percentage, and continuous data as median and range. The association between TNBC and other clinicopathologic variables and the significance of different prognostic markers were analyzed using the chi-squared test (or Fisher’s exact test when necessary). Association with survival was analyzed by the Kaplan-Meier plot and log-rank test. The Cox proportional hazards model was used to compute univariate and multivariate hazard ratios (HRs) for the study parameters with 95% confidence intervals (95% CIs). In all of the tests, a *p*-value of less than 0.05 was considered statistically significant. Cutoff values for different biomarkers included in this study were chosen before statistical analysis.

## 3. Results

The prognoses of 1036 patients with breast cancer who underwent surgery were analyzed retrospectively according to pathological subclassification. Among these, 190 (18.3%) were diagnosed with TNBC, and 846 (81.7%) with non-TNBC. Adjuvant chemotherapy was provided to 138/190 (72.6%) TNBC patients; 60 patients received an anthracycline-based regimen and 78 received a 5-FU-based regimen. Patients with TNBC had a significantly higher relapse rate compared to those with non-TNBC (*p* < 0.001, log-rank) (Supplemental Figure S1A). Furthermore, patients with TNBC also had a significantly poorer CSS rate than those with non-TNBC (*p* < 0.001, log-rank) (Supplemental Figure S1B).

**Figure 2:**
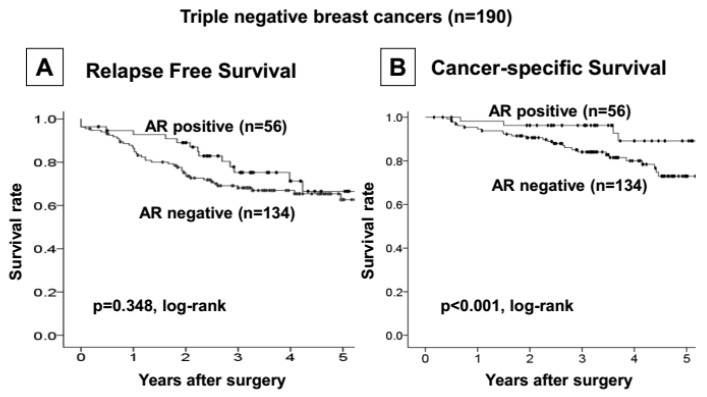
Cancer specific and relapse-free survival of patients based on AR expression in triple-negative breast cancers. AR expression cases had significantly good prognosis compared to the non-expression cases (**A**), but no significant difference in relapse-free survival rate was observed between AR-positive and negative triple-negative breast caluncer (TNBC) cases (**B**).

Fifty-six of 190 (29.5%) TNBC tumors expressed AR. No correlation was found between clinicopathologic characteristics and AR expression (Table 1). Additionally, no significant difference was observed in RFS rates between patients with AR-positive and -negative TNBC (*p* = 0.348, log-rank) (Figure 2A). However, the patients with AR-expressing tumors had significantly better prognoses than those with non-AR-expressing tumors (*p* < 0.001, log-rank) (Figure 2B). A statistical analysis of clinical factors demonstrated that advanced disease stage, tumor diameter ≥2 cm, positive axillary lymph node metastasis, higher histological grade, and negative tumor AR expression correlated significantly with poorer RFS. A multivariate analysis demonstrated that positive axillary lymph node metastasis was an independent and the strongest factor indicating higher risk of recurrence in patients with TNBC (*p* = 0.011, HR = 3.30). In addition, AR expression was found to be an independent factor indicating favorable prognosis in patients with TNBC (*p* = 0.039, HR = 0.36) (Table 2).

**Figure 3:**
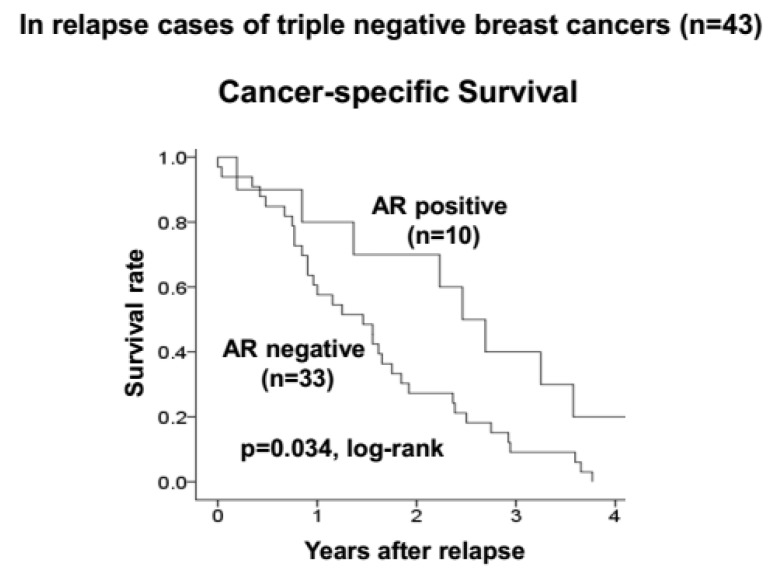
In relapse cases of TNBC. AR-positive TNBC had significantly good prognosis compared to negative cases.

Among 43 patients who suffered from disease relapse, 10 (23.3%) had AR-positive TNBC. When CSS after the relapse was investigated, patients with AR-positive TNBC had a significantly better prognosis than those with AR-negative TNBC (*p* = 0.034, log-rank) (Figure 3). However, there were no clinical features or pathological characteristics observed that may have influenced the increased survival rate in patients with AR-positive TNBC in comparison with those with AR-negative TNBC (Table 3).

## 4. Discussion

In recent studies, it has been determined that TNBC may further be classified into seven subtypes according to its gene expression profile [10,11], and the subtypes may respond differently to standardized therapeutic efforts [31]. According to previous studies, AR expression is commonly found in tumors that also express ER, and the prevalence of AR expression in TNBCs is reported less frequently, ranging from 13.7% to 64.3% (total 317/1227; 25.8%) [8,30,32,33,34]. This variability may be caused by differences in the techniques or criteria used to define AR positivity [8,29,30,32,33,34]. For AR positivity, many studies have adopted the standardized criteria for determining ER and PR positivity in breast cancer, defined as >1% positive cancer cells, which was also used in our study [29,30]. We found 30% of TNBCs expressed AR, which was in line with previous reports. Our study included as many as 190 TNBCs, although we did not examine the genetic profiles of each AR-positive tumor to determine which of these tumors could be classified into the luminal androgen receptor (LAR) subtype [30]. However, we did demonstrate that AR-positive TNBCs had different characteristics than AR-negative TNBCs. Thus, we believe that most of the AR-positive TNBCs could be categorized as the LAR subtype, and the population of the LAR subtype in TNBC would not be rare, as has been described by Lehmann et al. [10]. As with the luminal A and B (ER+) subtypes, overexpression of FOXA1 is observed as in LAR subtype TNBCs [10]. Breast tumors with FOXA1 overexpression have been reported to have a good prognosis, and we expect that the expression of FOXA1 will be tested in AR-positive TNBCs in the future.

As has been suggested in previous reports, we observed a significant difference in disease-free survival between patients with AR-positive and -negative TNBC [30,32,34]. Patients with AR-positive TNBC had disease recurrence later, by approximately 2 years, compared with those with AR-negative TNBC. Previous studies have shown that AR-positive tumors are associated with lower Ki-67 index [33], postmenopausal status, positive nodal status [30], higher tumor grade, and development of distant metastasis [8]. However, in our samples, the profiles of the patient or the initial disease did not differ between AR-positive and -negative TNBC. We also observed no difference in the site of recurrence (loco-regional or distant). These observations suggested that AR-positive TNBC has similar clinical characteristics to AR-negative TNBC. There have been consistent results concerning the difference in the population of TNBC and AR positivity according to race. AR-positive TNBCs in Japanese women may have unknown characteristics distinct from TNBCs as a whole that could contribute to the longer time to disease relapse. Signals generated by AR expression have been confirmed to display adverse effects on cellular proliferation in some breast cancer cell lines treated with 5-alpha-dihydrotestosterone [35]. These molecular mechanisms could be involved in delaying disease relapse.

AR expression also had a significant effect on CSS. Patients with AR-positive TNBCs survived longer after recurrence than those with AR-negative TNBCs. This clearly suggests a difference in malignant potential between AR-positive and -negative TNBC. However, we could not identify any specific factor responsible for this increase in survival. This was a retrospective study, and thus we could not alter the treatment strategies as the result of AR expression for any patient. Therefore, the difference in survival might be caused by differences in sensitivity to conventional treatments or by the innate nature of the AR-positive TNBC phenotype. Further investigation is required to identify the precise characteristics of AR-positive TNBCs. We have previously reported that TNBCs that are positive for AR expression have a significantly lower rate of pathological complete response (pCR) in neoadjuvant chemotherapy (NAC) and are chemotherapy-resistant [28].

A study examining treatment with AR antagonists in AR-expressing breast cancers is currently underway for clinical application [36]. There have been several agents that have been shown to have adverse effects on AR-positive cancer cells, and two clinical studies have already been conducted using targeted agents in AR-positive breast cancer [13]. The administration of bicalutamide to patients with metastatic AR-positive TNBC resulted in stable disease for 24 months in 19% of patients. These agents could become important treatment options for AR-positive TNBC in the near future [37].

In this study, we carried out protein expression analysis on TNBCs using immunohistochemical staining and investigated the clinical significance of AR expression. Although we observed no significant difference in the RFS rate between AR-positive and -negative TNBC cases, many late relapse cases (4 or more years to recurrence) showed luminal type relapse. Thus, we believe that AR-positive TNBC has biological properties different from those of the basal-like TNBCs that display a high degree of malignancy, and that are more similar to the hormone receptor-positive luminal subtypes. We believe that among the TNBC subtypes, the biological malignancy of AR-positive TNBC is lower than other subtypes.

## 5. Conclusions

We conclude that AR expression may be useful as a subclassification marker for good prognosis in TNBC, and that AR-positive TNBCs may be responsive to anti-androgen endocrine therapy.

## Figures and Tables

**Table 1:** Correlation between clinicopathological features and androgen receptor expression in 190 triple-negative breast cancer.

Parameters	Androgen Receptor		*p* Value
	Positive (*n* = 56)	Negative (*n* = 134)	
Age at operation			
≤55	27 (48.2%)	57 (42.5%)	
>55	29 (51.8%)	77 (57.5%)	0.473
Stage			
1	16 (28.6%)	43 (32.1%)	
2–4	40 (71.4%)	91 (67.9%)	0.633
Tumor size (cm)			
≤2	21 (37.5%)	54 (40.3%)	
>2	35 (62.5%)	80 (59.7%)	0.719
Lymph node status			
Negative	34 (60.7%)	81 (60.4%)	
Positive	22 (39.3%)	53 (39.6%)	0.973
Lymphatic invasion			
Negative	44 (78.6%)	91 (67.9%)	
Positive	12 (21.4%)	43 (32.1%)	0.140
Vascular invasion			
Negative	56 (100.0%)	129 (96.3%)	
Positive	0 (0%)	5 (3.7%)	0.171
Histologic type			
IDC	48 (85.6%)	116 (86.6%)	
Special type	8 (14.3%)	18 (13.4%)	0.876
Histological grade			
1–2	28 (50.0%)	55 (41.0%)	
3	28 (50.0%)	79 (59.0%)	0.257
Ki67			
Negative	24 (42.9%)	57 (42.5%)	
Positive	32 (57.1%)	77 (57.5%)	0.968

IDC, invasive ductal carcinoma.

**Table 2:** Univariate and multivariate analysis with respect to progression free survival in 190 triple-negative breast cancers.

Parameters	Univarite Analysis			Multivariate Analysis		
	Hazard Ratio	95% CI	*p* Value	Hazard Ratio	95% CI	*p* Value
Androgen receptor	0.34	0.13–0.87	0.025	0.36	0.14–0.95	0.039
Positive vs. Negative						
Pathological stage	2.54	1.04–6.22	0.041	0.40	0.62–2.54	0.329
I vs. II and III						
Tumor size (cm)	2.46	1.11–5.45	0.027	2.71	0.63–11.77	0.183
≤2 vs. >2						
Lymph node status	3.39	1.67–6.88	0.001	3.30	1.32–8.25	0.011
n0 vs. n1, n2, n3						
Lymphatic invasion	1.94	0.99–3.75	0.054	1.23	0.65–2.66	0.565
ly0 vs. ly1, ly2, ly3						
Histological grade	2.36	1.01–5.21	0.034	1.78	0.79–4.01	0.162
1, 2 vs. 3						

**Table 3:** Correlation between clinicopathological features and androgen receptor expression among 43 relapsed cases in 190 triple-negative breast cancer.

Parameters	Androgen Receptor		*p* Value
	Positive (*n* = 10)	Negative (*n* = 33)	
Age at operation			
≤55	5 (50.0%)	20 (60.6%)	
>55	5 (50.0%)	13 (39.4%)	0.551
Stage			
1	2 (20.0%)	8 (24.2%)	
2–4	8 (80.0%)	25 (75.8%)	0.575
Tumor size (cm)			
≤2	3 (30.0%)	9 (27.3%)	
>2	7 (70.0%)	24 (72.3%)	0.579
Lymph node status			
Negative	5 (50.0%)	14 (42.4%)	
Positive	5 (50.0%)	19 (57.6%)	0.673
Lymphatic invasion			
Negative	6 (60.0%)	14 (42.4%)	
Positive	4 (40.0%)	19 (57.6%)	0.269
Vascular invasion			
Negative	10 (100.0%)	31 (93.9%)	
Positive	0 (0%)	2 (6.1%)	0.585
Histologic type			
IDC	10 (100.0%)	28 (84.8%)	
Special type	0 (0.0%)	5 (15.2%)	0.247
Histological grade			
1–2	3 (30.0%)	8 (24.2%)	
3	7 (70.0%)	25 (75.8%)	0.504
Ki67			
Negative	1 (10.0%)	11 (33.3%)	
Positive	9 (90.0%)	22 (66.7%)	0.149
Relapse and metastases			
Locoregional	6 (60.0%)	19 (57.6%)	
Distant	4 (40.0%)	14 (42.4%)	0.594

IDC, invasive ductal carcinoma.

## Supplementary Materials

The following are available online at http://www.mdpi.com/2072-6694/9/1/4/s1. Figure S1: Correlation between the triple-negative phenotype and cancer specific survival and relapse-free survival. The patients with triple-negative breast cancers had a significantly poorer outcome in all breast cancers (A,B).
