# Supplementary material for: Expression and Clinical Significance of Androgen Receptor in Triple-Negative Breast Cancer
Source: Cancers (Basel). 2017 Jan 6;9(1):4. doi: 10.3390/cancers9010004 (PMC5295775; doi:10.3390/cancers9010004)
Supplement: Supplementary file 1 [file cancers-09-00004-s001.pdf]

Supplementary Materials: Expression and Clinical Significance of Androgen Receptor in Triple-Negative Breast Cancer

Yuka Asano, Shinichiro Kashiwagi, Wataru Goto, Sayaka Tanaka, Tamami Morisaki, Tsutomu Takashima, Satoru Noda, Naoyoshi Onoda, Masahiko Ohsawa, Kosei Hirakawa and Masaichi Ohira

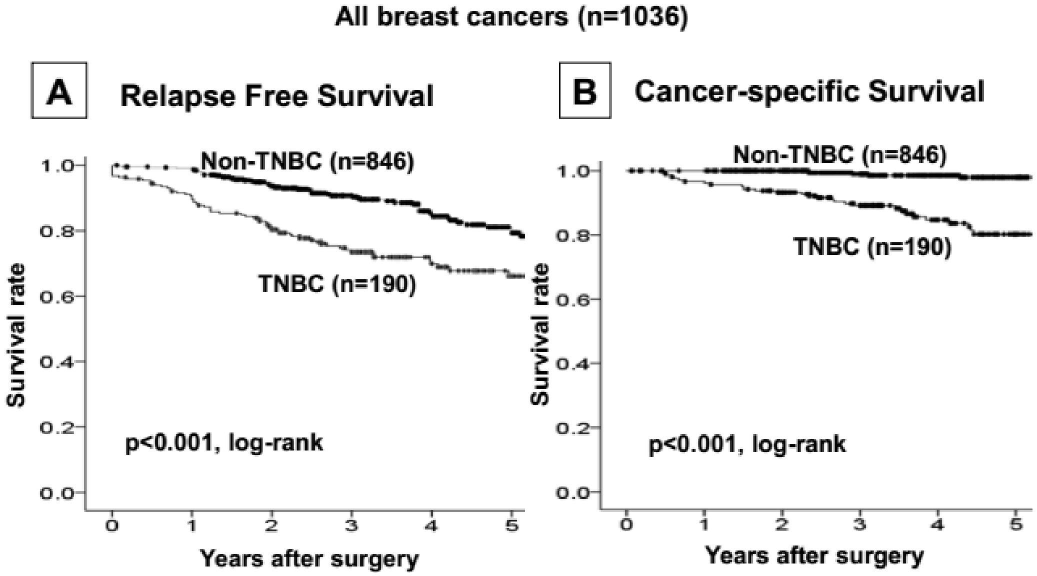

Figure S1. Correlation between the triple-negative phenotype and cancer specific survival and relapse-free survival. The patients with triple-negative breast cancers had a significantly poorer outcome in all breast cancers (**A**,**B**).
